# Linear energy transfer optimization mitigates biological model discrepancies in carbon ion radiotherapy for pelvic osteosarcoma

**DOI:** 10.1016/j.phro.2026.101113

**Published:** 2026-10-23

**Authors:** Natalia Sycheva, Katharina Kozyra, Katharina Seidensaal, Hanna Waldsperger, Semi B. Harrabi, Jürgen Debus, Andrea Mairani, Thomas Tessonnier

**Affiliations:** a Heidelberg Ion-Beam Therapy Center (HIT), Department of Radiation Oncology, Heidelberg University Hospital, Heidelberg, 69120, Germany; b Clinical Cooperation Unit Radiation Oncology, German Cancer Research Center (DKFZ), Heidelberg, 69120, Germany; c Department of Radiation Oncology, Heidelberg University Hospital, Heidelberg, 69120, Germany; d National Center for Tumor Diseases (NCT), Heidelberg University Hospital, Heidelberg, 69120, Germany; e ROCC Radiation Oncology Leonberg, Leonberg, Germany; f Heidelberg Institute of Radiation Oncology (HIRO), Heidelberg, 69120, Germany; g German Cancer Consortium (DKTK), Core Center Heidelberg, Heidelberg, 69120, Germany; h Clinical Cooperation Unit Translational Radiation Oncology, German Cancer Research Center (DKFZ), Heidelberg, 69120, Germany; i Centro Nazionale di Adroterapia Oncologica (CNAO), Pavia, 27100, Italy

**Keywords:** Carbon ion radiotherapy, LET optimization, Radiobiological model, LEM-I, mMKM, Osteosarcoma

## Abstract

**Background and purpose:**

In the combined treatment of osteosarcoma with a carbon ion radiotherapy (CIRT) boost, large pelvic tumors are associated with significantly lower tumor control compared to craniofacial localization. This could be linked to lower dose-averaged linear energy transfer (LET_d_) values in the target and larger uncertainties related to the model used for relative biological effectiveness (RBE) calculation. This study aimed to bridge the difference between RBE-weighted doses from two clinically used models, the local effect model I (LEM-I) and the modified microdosimetric kinetic model (mMKM), using LET_d_ optimization.

**Materials and methods:**

Fifteen pelvic osteosarcoma CIRT plans, initially optimized with LEM-I, were re-optimized to achieve higher target LET_d_ while preserving target and organ at risk (OAR) dose objectives. Both dose- and LET-optimized plans were recalculated with mMKM, and dose metrics were compared across the groups. Robustness against setup and range uncertainties and changes in patient anatomy (using control CTs) was investigated.

**Results:**

LET_d_ optimization resulted in target LET_d_ escalation without compromising LEM-I target coverage. The LET-optimized group also demonstrated better mMKM dose coverage and a significant decrease (up to 9% of prescription) in the disparity between LEM-I and mMKM target dose. OAR doses remained largely unaffected by model choice or optimization strategy. The robustness loss with LET_d_ optimization was minor (intra-patient dose variability increased by 0.3–2.3%) compared to the reduction in model-to-model variation.

**Conclusions:**

When adequately balanced with physical uncertainties, LET_d_ optimization could become a useful strategy for bridging RBE model predictions and increasing the efficacy of CIRT.

## Highlights

• Increased target linear energy transfer (LET) affected biological dose predictions.
• LET optimization did not compromise target coverage or organ at risk sparing.
• LET-optimized plans were more uniform upon recalculation with a different model.
• Model difference was reduced by up to 9% of prescription with LET optimization.
• LET optimization increased dose variability in robust evaluation by 0.3–2.3%.

## Graphical abstract

**Figure:**
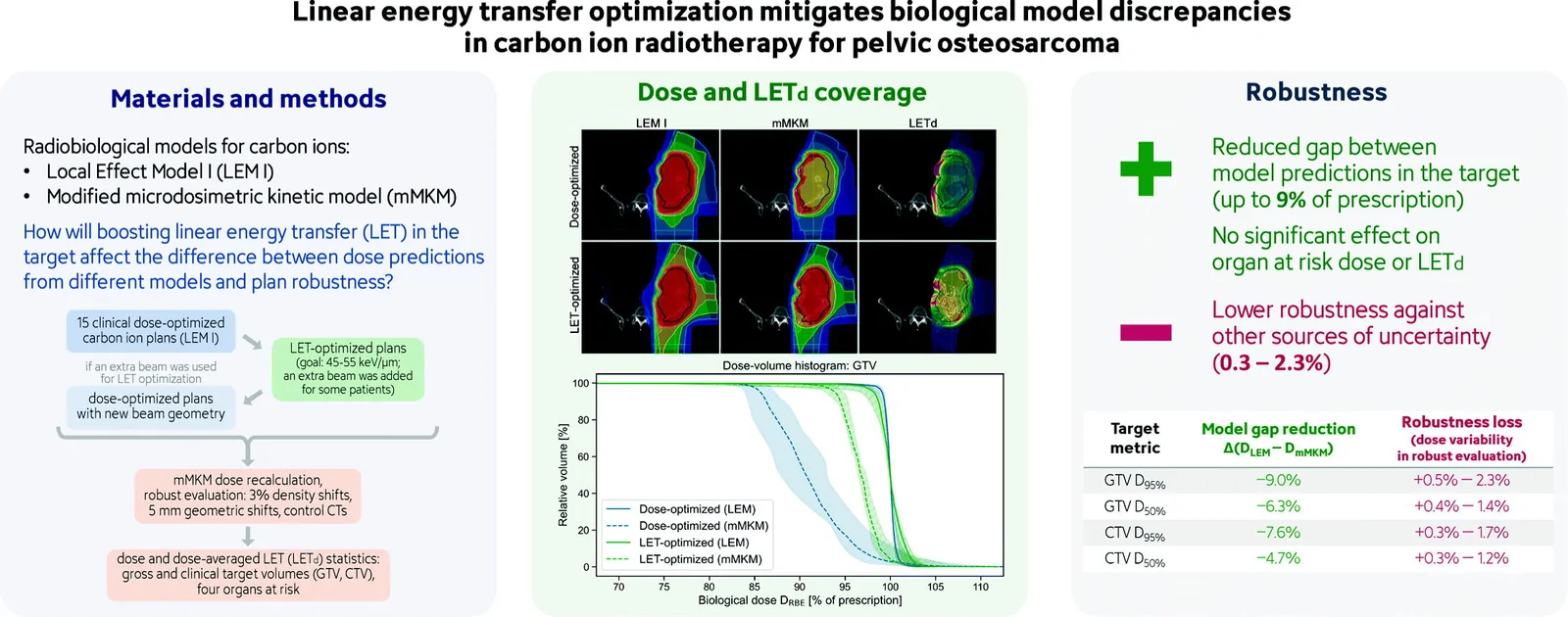

## 1 Introduction

Osteosarcoma is the most prevalent primary bone malignancy among children and adolescents [1]. While osteosarcoma of the extremities, typically treated with chemotherapy and surgery, has a 5-year event-free survival rate of 60–70% for non-metastatic disease [2], axial tumor location is associated with challenges in surgical treatment and an overall poorer prognosis [3], [4], highlighting the need for other therapeutic approaches.

Carbon ion radiation therapy (CIRT) stands out among radiation therapy modalities due to its advantageous depth dose characteristics and radiobiological effects associated with the high linear energy transfer (LET) near the end of particle trajectory, allowing for increased normal tissue sparing while maintaining target coverage compared to photon or proton beam irradiation [5]. CIRT alone or in combination with other modalities has been established as an alternative to surgery for locally advanced osteosarcoma, with fewer adverse events and favorable overall survival and local control [6], [7]. However, a number of challenges remain, especially pertaining to the treatment of large tumors, which have demonstrated less favorable outcomes [8].

It is increasingly evident that target dose coverage alone may not be the sole predictor of tumor control in CIRT. Higher near-minimum dose-averaged LET (LET_d_) in the target is associated with lower recurrence rates in pancreatic cancer [9], [10], unresectable chondrosarcoma [11], and sacral chordoma [12], [13]. The main benefit of LET_d_ escalation is high-LET radiation's ability to overcome hypoxia-induced radioresistance, with noticeable LET effects on the oxygen-enhancement ratio observed above the 50–60 keV/μm threshold [14], [15]; this is especially relevant for large tumors, where hypoxic cores are more likely to form.

With treatment optimization strategies based on biologically weighted dose, carbon ions demonstrate a highly non-uniform LET distribution, with its maximum at the distal edge of the spread-out Bragg peak. The LET_d_ distribution will thus also depend on plan geometry (the number of beams and their angles) and tumor size: in larger tumors, most of the target dose is delivered in the low-to-medium LET range, essentially failing to utilize the LET advantage of heavy ions [16].

Another consequence of non-uniform LET distributions is the large spatial variation in relative biological effectiveness (RBE) along the particle track, meaning that the choice of radiobiological model for RBE calculation can drastically change the biological dose distribution and thus the treatment outcome. Historically, European heavy ion therapy facilities have employed the local effect model I (LEM-I) [17] to model the RBE in CIRT; it is known, however, that LEM-I tends to overestimate RBE at low and mid-range LET and may also slightly underestimate it at higher LET [18]. In large tumors, where relatively low LET is observed in most of the volume, LEM-I will therefore likely overestimate the RBE-weighted dose in the central part of the target. On the contrary, the modified microdosimetric kinetic model (mMKM) [19] has been shown to be in better agreement with both in vitro and in vivo data compared to LEM-I [20]. Within a single CIRT plan without any LET_d_-boosting strategies, differences of up to 15–20% can be observed between the biological dose predictions of LEM-I and mMKM [20], [21], [22].

However, despite the advantages of mMKM for CIRT planning, the potential transition away from LEM-I is a gradual process requiring great care and consideration [23]. Most existing European protocols rely on LEM-based prescriptions and constraints, whose translation into mMKM is not straightforward and further complicated by the mMKM used at the National Institute of Radiological Sciences in Japan (NIRS-mMKM [19]) not being directly comparable to the mMKM variant used at the Heidelberg Ion-Beam Therapy Center (HIT-mMKM) (Supplementary Material A). The choice of radiobiological model and corresponding clinical dose constraints is also a timely question for new centers planning to start CIRT in the upcoming years [24], [25]. In this context, as the differences between LEM and mMKM are largest in low-LET_d_ regions [20], target LET_d_ optimization could bring in the additional advantage of reducing the uncertainties related to the choice of radiobiological model.

The aim of this study was to assess the impact of LET_d_ optimization of LEM-I plans on the differences in biological dose predictions from LEM-I and mMKM, with the intention of potentially enhancing the efficacy of CIRT for unresectable osteosarcoma in clinical practice.

## 2 Materials and methods

### 2.1 Patient cohort

The treatment planning study was conducted for fifteen patients with unresectable pelvic osteosarcoma who received combined ion-beam radiation therapy at the Heidelberg Ion-Beam Therapy Center (HIT) according to the protocol for the OSCAR trial [8]. The study was conducted in accordance with the Declaration of Helsinki and approved by the Ethics committee of Heidelberg University (S-488/2024).

The treatment involved a primary proton plan of 54 Gy(RBE)^1^ (RBE = 1.1) in 27 fractions and a CIRT boost plan of 18 Gy(RBE) (LEM-I) in 6 fractions, with prescription dose D_p_ = 18 Gy(RBE) defined as the median dose to the clinical target volume (CTV); for the purpose of this study, only the CIRT boost plans were evaluated. The median gross tumor volume (GTV) was 411.3 cm^3^ (interquartile range (IQR): 193.3–686.1 cm^3^). CTVs were obtained by adding an isotropic margin of 3–7 mm, in accordance with current clinical practice [8], [26]. The planning target volume (PTV) was defined as the CTV plus margins of 7 mm in beam directions and 5 mm in the remaining directions.

### 2.2 Initial treatment plan and LET optimization strategy

The initial plans were designed and optimized within the RayStation 10 A treatment planning system (TPS) (RaySearch Laboratories, Sweden); for this study, the doses were recalculated with RayStation 2024B (carbon pencil-beam scanning engine version 7.1), where all subsequent re-optimizations and investigations were performed. For plan design and optimization, a uniform 2 mm dose grid and a hexagonal spot pattern were used, with 3.6 mm spot spacing and 3.1 mm energy layer spacing. RBE dose was calculated with LEM-I, with α/β = 2 Gy (where $\alpha_{x}$ = 0.1 Gy^−1^, $\beta_{x}\;$= 0.05 Gy^−2^), $D_{t}$ = 30 Gy, and $r_{n}$ = 5 μm [20]. Depending on tumor volume and location, initial plans required one (*n* = 2), two (*n* = 10) or three (*n* = 3) beams (Supplementary Table S1).

Target LET_d_ objectives of 45–55 keV/μm (Table 1) were based on existing clinical evidence for other indications [10], [11], [12] and a simulation study conducted in the TPS determining the isoRBE from LEM-I and mMKM as a function of LET_d_ (Supplementary Fig. S1). Constraints on doses to organs at risk (OARs) were defined for each patient individually to adhere to the total dose limits for the entire course of treatment (including the primary proton plan).

**Table 1:** Main dose objectives (for all plans) and LET_d_ objectives (for LET-optimized plans) for the target specified in the optimization function. GTV − 10 mm is the GTV minus a uniform 10-mm margin.

Primary prescription (D_p_)	CTV D_50%_ = 18 Gy(RBE) / 6 fractions
Target coverage objectives	
GTV	Min. dose >17.1 Gy(RBE) = 95% D_p_
CTV	Uniform dose 18 Gy(RBE)
PTV	Max. dose <18.75 Gy(RBE) = 104% D_p_

LET_d_ optimization objectives	
CTV	Min. LET_d_ > 45 keV/μm
GTV	Min. LET_d_ > 50 keV/μm
GTV − 10 mm	Min. LET_d_ > 55 keV/μm

For LET-optimized plans, after initial spot positioning on the PTV only, robust optimization against ±7 mm setup uncertainties (a total of fifteen scenarios including intermediate ±4 mm shifts) was added to the existing target volume objectives (minimum dose for GTV and CTV, maximum dose for PTV). If the LET_d_ objective was not achievable with the original beam setup, an additional beam was introduced. In such cases, to account for and evaluate potential changes in LET_d_ distributions and plan robustness, a purely dose-optimized plan with the new number of beams (DoseOpt) was also created.

Following LEM-I-based optimization, dose distributions were recalculated using mMKM with α/β = 2 Gy ($\alpha_{0}$ = 0.05 Gy^−1^, $\beta_{x}\;$= 0.025 Gy^−2^), $r_{d}$ = 0.3 μm, and $r_{n}$ = 3.6 μm [27]. No additional mMKM-based re-optimization was performed afterwards so as to compare the RBE models' predictions for otherwise identical plans.

### 2.3 Treatment plan evaluation

Dose coverage of the target was evaluated using the corresponding dose-volume histogram (DVH) metrics and, in particular, doses received by at least 95% (D_95%_), 50% (D_50%_) and 2% (D_2%_) of the target volumes; LET_d_ distribution was assessed by examining LET_d_ volume histograms (LVH), LET_95%_ and LET_50%_ in the target volumes, as well as relative volumes meeting the optimization objectives (V_50keV/μm_ for the GTV, V_45keV/μm_ for the CTV).

To assess the doses to OARs (bowel, cauda equina, rectum and sigmoid colon), near-maximum dose defined as dose received by at least 0.03 cm^3^ of the organ volume ($D_{0.03{\mathrm{cm}}^{3}}$) was used. The LET_d_ distribution was evaluated using LET_50%_ and LET_2%_ with a dose threshold of 9 Gy(RBE) (50% of D_p_).

To find statistically significant differences between groups of interest, the two-sided Wilcoxon signed-rank test (α = 0.05) was used. Quantitative analysis of the data was performed using the Pingouin 0.5.5 Python package.

### 2.4 Robust evaluation

Dose-volume metrics of LEM-I plans were assessed with respect to two robust optimization scenario groups: ±3% density shifts (two scenarios) and ± 5 mm geometric isocenter shifts along the three main axes and the diagonals (fourteen scenarios, Supplementary Fig. S2). Density and geometric shifts were assessed independently of each other, i.e., only one type of uncertainty was applied at a time in each scenario.

To assess robustness against changes in patient anatomy, the plans were also recalculated on additional CT scans available for each patient (from two to six control scans acquired during treatment).

For each of the three robustness aspects, the standard deviation (SD) of $D-D_{\mathrm{ref}}$ within a single patient was used as a measure of statistical dispersion, where D was the robust scenario or evaluation dose metric and $D_{\mathrm{ref}}$ was the corresponding initial plan dose metric. The cohort medians of these intra-patient standard deviations were then compared between the dose-optimized and LET-optimized groups using the same statistical methods as described in 2.3. For each patient, the change in these SDs between dose-optimized and LET-optimized plans was also calculated and the cohort median difference was compared to the simultaneous effect on model-to-model dose difference in the nominal plan.

## 3 Results

LET-optimized plans were created for each reference plan; to achieve an optimal LET_d_ distribution, twelve out of fifteen cases required an additional beam (Supplementary Table S1), for which a second dose-optimized plan with the new beam configuration was created. Fig. 1 and Supplementary Fig. S3 illustrate dose and LET_d_ distributions for the patients with the largest GTV and the median GTV of the cohort, respectively.

**Fig. 1:**
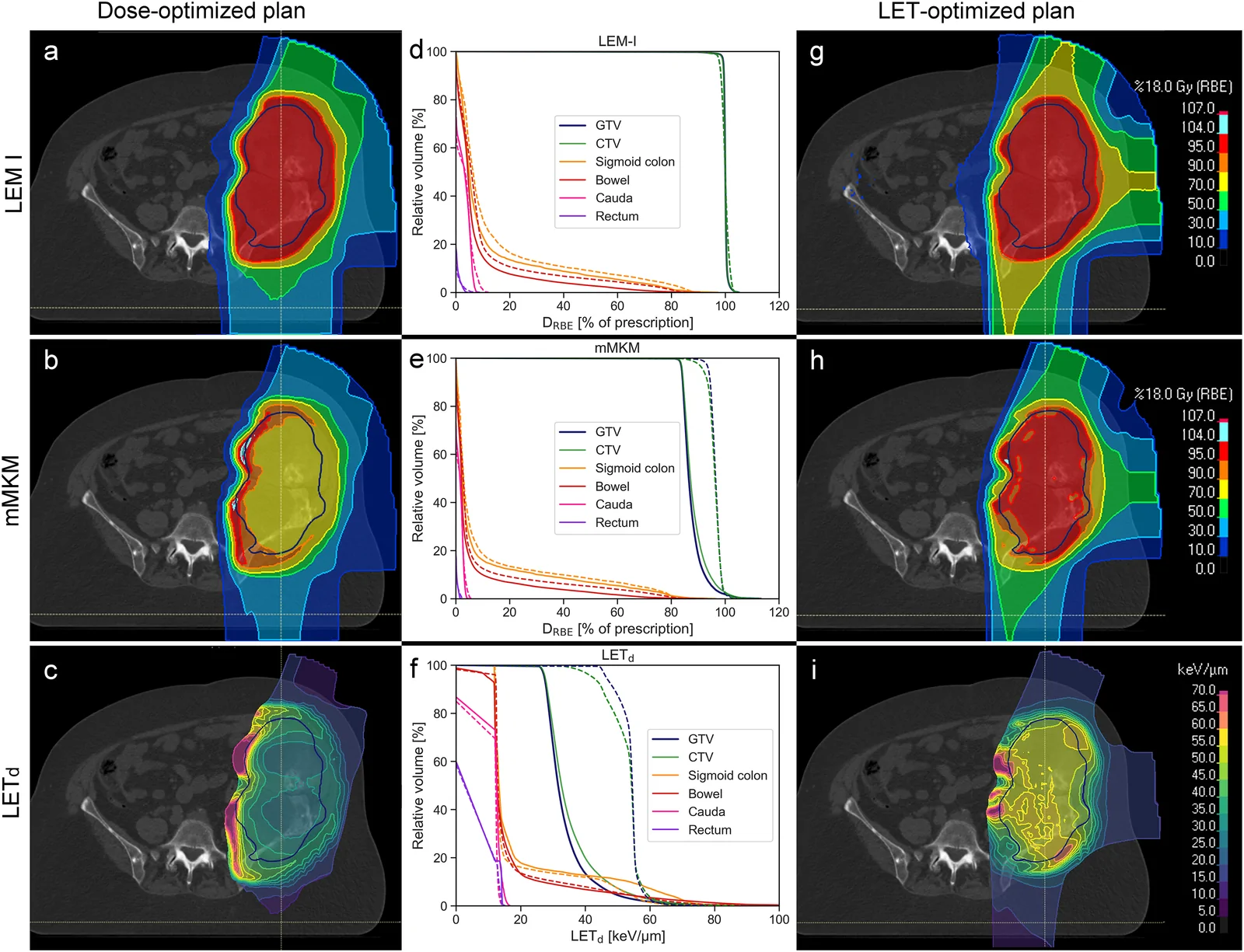
Axial slices of the original (LEM-I-based) biological dose, mMKM-recalculated biological dose, and LET_d_ distributions for the dose-optimized (a-c) and LET-optimized (g-i) plans (identical beam geometries) as well as the corresponding dose and LET_d_ volume histograms (d-f; solid lines: dose-optimized plan; dashed lines: LET-optimized plan) in the target and four organs at risk in a single patient case (V_GTV_ = 1187.4 cm^3^). The gross tumor volume is delineated in blue. (For interpretation of the references to colour in this figure legend, the reader is referred to the web version of this article.)

When a change in beam configuration was necessary, minor but statistically significant differences between the dose-optimized plans with and without an additional beam (DoseOpt and Initial, respectively) were only observed for LEM-I D_95%_ in the GTV and CTV (a median cohort increase of 0.5% and 1.2% respectively with an additional beam, *p* = 0.003); other dose and LET_d_ metrics were not significantly affected. All further comparisons between the dose- and LET-optimized plans were made using the DoseOpt plans to exclude potential effects of beam geometry.

In terms of LET_d_ distribution, most LET-optimized plans did not meet the minimum target LET_d_ objectives set in the optimization function: the median V_50keV/μm_ in the GTV and V_45keV/μm_ in the CTV were only equal to 88% and 97% of the respective volumes, although this was still an improvement compared to the initial values of 15% and 39% (Supplementary Table S2). Overall, using LET_d_ optimization led to a significant increase in LET_95%_ and LET_50%_ in the target (*p* < 0.001), with the median LET_95%_ in the GTV increasing from 31 keV/μm (IQR: 28–33 keV/μm) to 49 keV/μm (IQR: 47–50 keV/μm).

Adequate LEM-I target coverage was achieved in both groups, with no significant differences observed between the dose-optimized and LET-optimized plans. This was not the case for mMKM-recalculated plans: the primary prescription of D_50%_ = D_p_ = 18 Gy(RBE) in the CTV was not satisfied in any of the plans, with a median of 16.5 Gy(RBE) (IQR: 16.2–16.8 Gy(RBE)) in the dose-optimized group and 17.3 Gy(RBE) (IQR: 17.3–17.4 Gy(RBE)) in the LET-optimized group. With regard to the (near-)minimum GTV dose objective, LET-optimized plans also performed better, with the median D_95%_ = 94% (IQR: 93–95%) of D_p_ as opposed to 86% (IQR: 83–86%) in the initial plans.

Importantly, the use of LET_d_ optimization significantly reduced the disparity between LEM-I and mMKM's predictions compared to dose-optimized plans (Fig. 2), with the exception of near-maximum dose D_2%_, where no significant differences were observed. The effect was particularly apparent in mMKM cold spots, which generally corresponded to the low-LET regions of dose-optimized plans, with a median reduction in model difference of 9.0% of prescription for D_95%_ and 6.3% for D_50%_ in the GTV with LET_d_ optimization (*p* < 0.001).

**Fig. 2:**
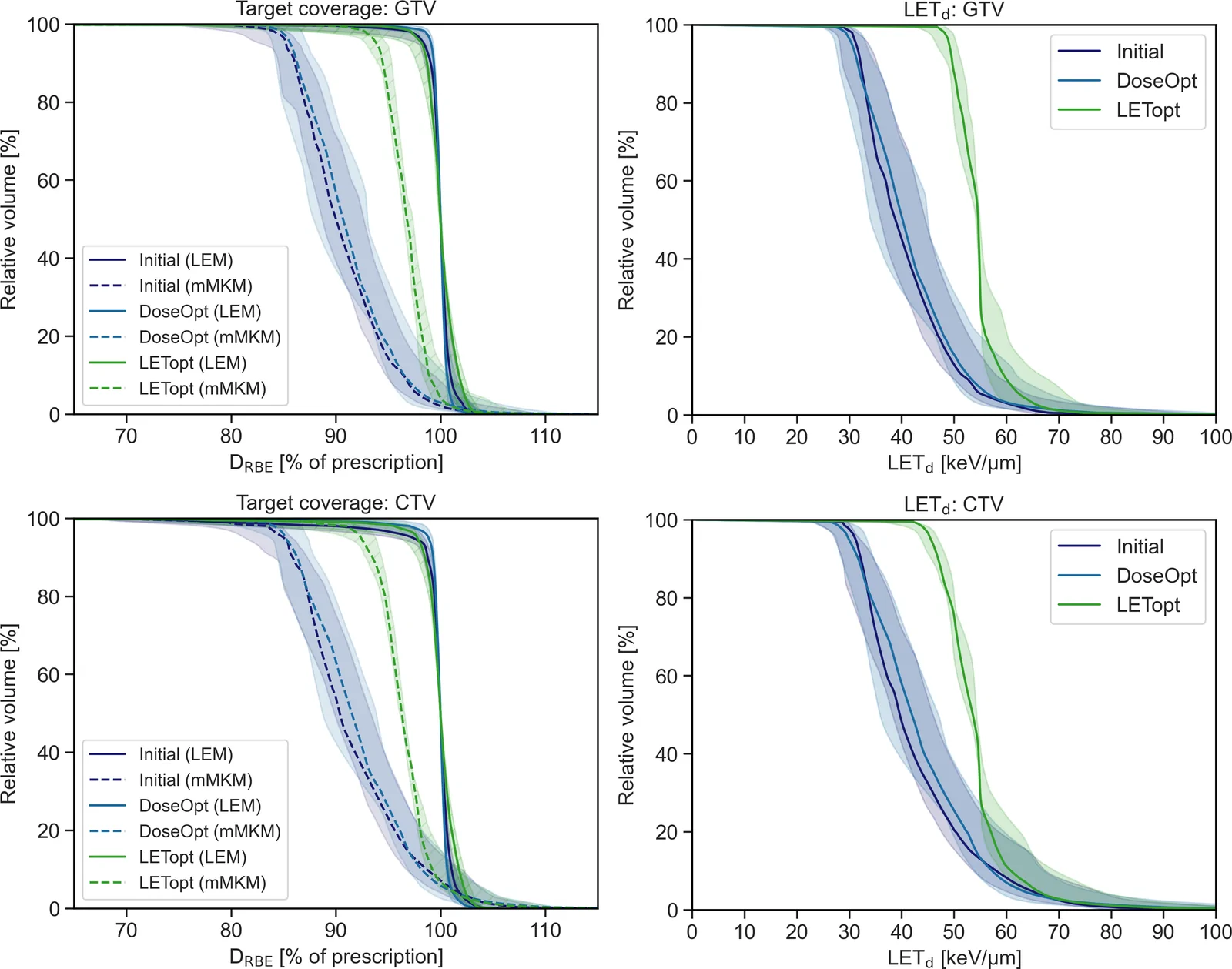
Cohort median LEM-I (solid) and mMKM (dashed) dose and LET_d_ volume histograms for the GTV and CTV. Initial = dose-optimized, original beam configurations; DoseOpt = dose-optimized, beam geometry identical to corresponding LET-optimized plans; LETopt = LET-optimized plans. The shaded areas represent the cohort interquartile range. The reference prescription dose is 18 Gy(RBE).

In contrast, LET_d_ distributions in the OARs were not substantially affected by LET_d_ optimization. Similarly, neither the optimization strategy nor the radiobiological model had a significant effect on near-maximum RBE dose (Fig. 3).

**Fig. 3:**
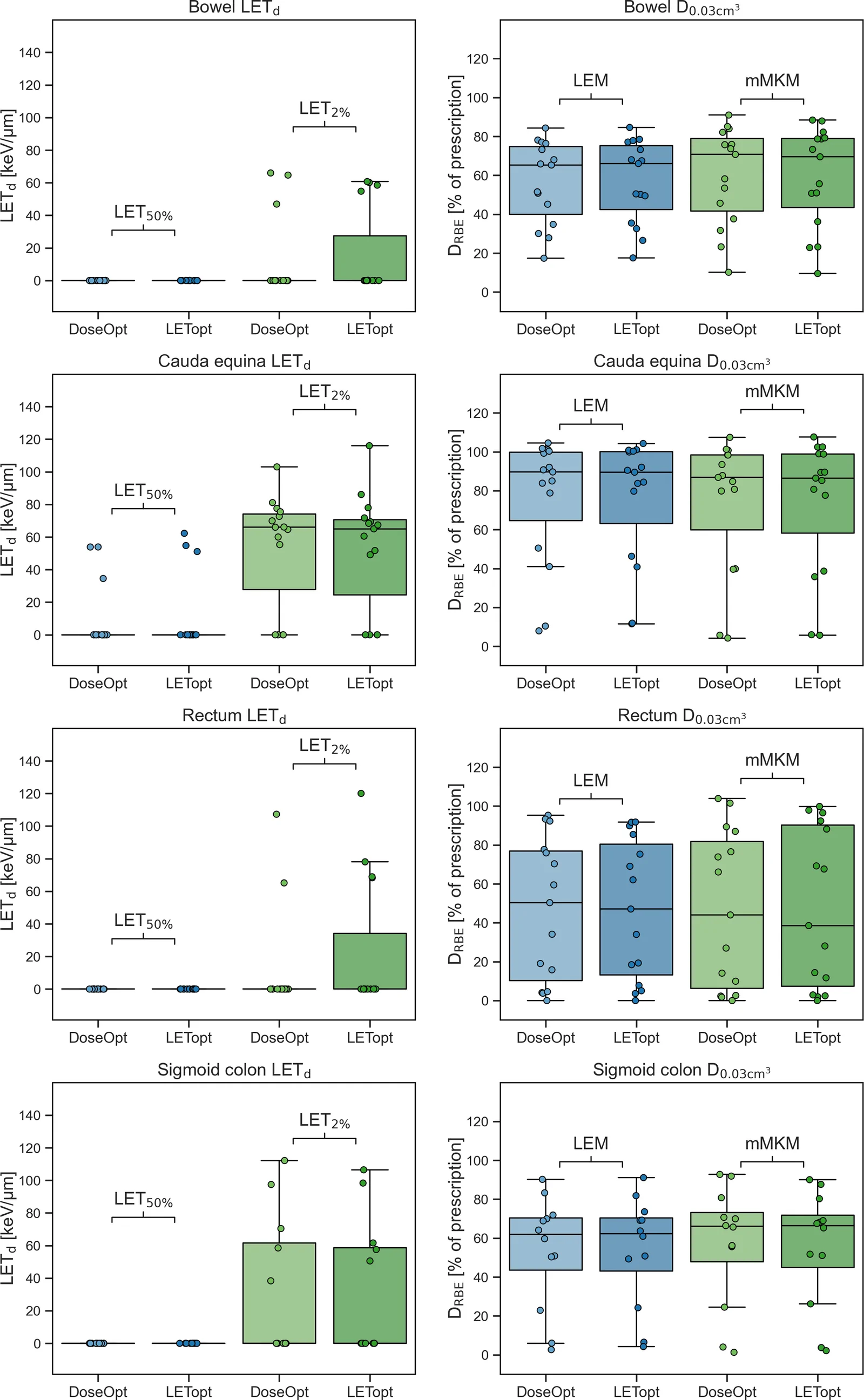
LET_2%_, LET_50%_ and near-maximum biological dose ($D_{0.03{\mathrm{cm}}^{3}}$) in four organs at risk for dose-optimized (DoseOpt) and LET-optimized (LETopt) plans with identical beam geometries. The reference prescription dose is 18 Gy(RBE); LET_d_ is presented with a dose threshold of 50% of the prescription.

From robust evaluation, it was apparent that even though large statistically significant differences were not observed in every case, LET_d_ optimization did indeed worsen plan robustness in terms of target coverage, with the spread of data increasing for almost all target metrics and scenario groups (Fig. 4). The observed negative effects were particularly notable in the 3% density shift robust analysis groups, with statistically significant increases in dose-volume metric dispersion of 0.7–2.3% (*p* < 0.05) across all target metrics (Table 2). On the other hand, doses to OARs (Supplementary Fig. S4) did not display a strong trend in either direction.

**Fig. 4:**
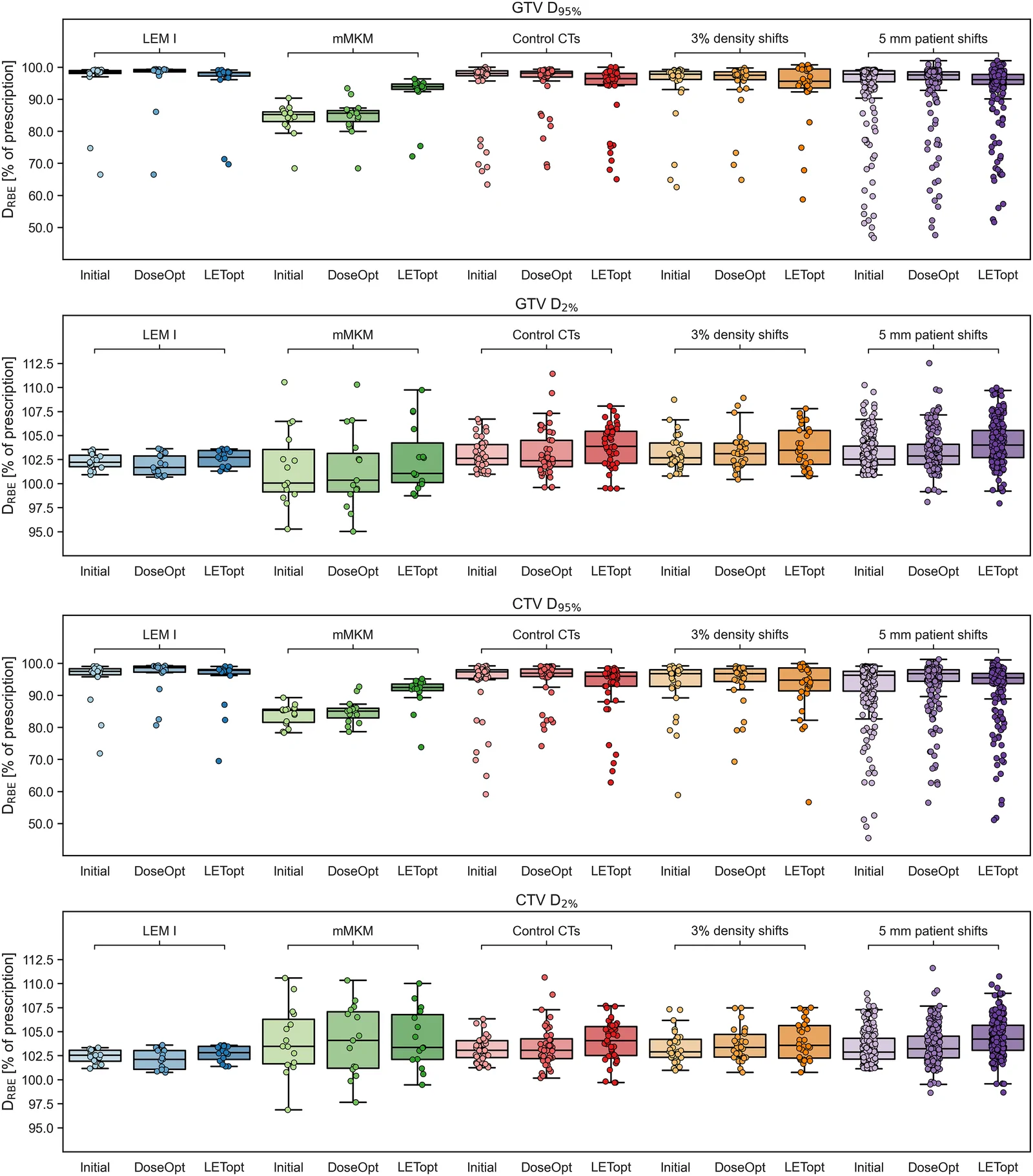
Evaluation of D_95%_ and D_2%_ in the GTV and the CTV with dose-based optimization and LET optimization aimed to achieve a minimum LET_d_ of 50 keV/μm in the GTV and a minimum LET_d_ of 45 keV/μm in the CTV. Initial = dose-optimized, original beam configurations; DoseOpt = dose-optimized, beam geometry identical to corresponding LET-optimized plans; LETopt = LET-optimized. LEM-I plans were additionally evaluated with respect to control CTs acquired during treatment, 3% density shifts (2 scenarios) and 5 mm geometric shifts (14 scenarios). The reference prescription dose is 18 Gy(RBE).

**Table 2:** Comparison of the effects of LET_d_ optimization on the disparity between RBE model predictions (D_LEM_ – D_mMKM_) and plan robustness, assessed as the standard deviation (SD) of D − D_ref_, where D_ref_ is the initial LEM plan dose. Model-to-model dose difference, standard deviations across robustness scenarios, and their resulting change from dose- to LET-optimized plans (Δ) were first calculated separately for each patient; then the median values and interquartile ranges (IQR) of Δ were obtained. A negative value indicates reduction in dose difference or SD with LET_d_ optimization. cCT – control CTs; 3% and 5 mm – robust optimization scenario groups of 3% density shifts and 5 mm geometric shifts. The reference prescription dose is 18 Gy(RBE). Differences between the groups were investigated using the two-sided Wilcoxon signed-rank test, with the significance threshold of α = 0.05. * *p* < 0.05, ** *p* < 0.01, *** *p* < 0.001.

	Change from dose-optimized to LET-optimized plan, median (IQR)			
	Δ(D_LEM_ − D_mMKM_) [% 18 Gy(RBE)]	ΔSD(D − D_ref_) (cCT, LEM) [% 18 Gy(RBE)]	ΔSD(D − D_ref_) (5 mm, LEM) [% 18 Gy(RBE)]	ΔSD(D − D_ref_) (3%, LEM) [% 18 Gy(RBE)]
**Target**				
GTV D_95%_	−9.0*** (−10.9; −7.9)	+0.5* (−0.1; +0.7)	+0.7 (+0.3; +1.0)	+2.3*** (+1.2; +2.5)
GTV D_50%_	−6.3*** (−7.5; −4.3)	+0.4*** (+0.3; +0.7)	+1.2*** (+0.5; +1.3)	+1.4*** (+0.7; +1.8)
GTV D_2%_	+0.1 (−1.1; +0.6)	+0.3 (+0.0; +1.1)	+0.9** (+0.4; +1.1)	+0.7* (+0.6; +1.6)
CTV D_95%_	−7.6*** (−9.6; −5.9)	+0.5** (+0.3; +0.6)	+0.3 (−0.2; +0.6)	+1.7** (+1.3; +2.1)
CTV D_50%_	−4.7*** (−6.1; −2.9)	+0.3*** (+0.2; +0.6)	+0.9*** (+0.3; +1.1)	+1.2*** (+0.4; +1.4)
CTV D_2%_	+0.2 (+0.1; +1.1)	+0.1 (−0.1; +1.0)	+0.5** (+0.2; +0.9)	+0.7** (+0.6; +1.4)

**Organs at risk**				
Bowel $D_{0.03{\mathrm{cm}}^{3}}$	+2.0 (−0.2; +3.6)	−0.3 (−2.0; +0.9)	−0.7 (−1.6; −0.3)	−1.9* (−4.6; +0.0)
Cauda equina $D_{0.03{\mathrm{cm}}^{3}}$	+0.1 (−0.8; +1.7)	−0.1 (−0.5; +1.1)	+0.1 (−0.6; +1.2)	+0.7 (−0.2; +1.2)
Rectum $D_{0.03{\mathrm{cm}}^{3}}$	+0.0 (−0.9; +1.4)	−0.1 (−0.7; +0.4)	−0.3 (−1.2; +0.1)	+0.5 (−0.0; +2.2)
Sigmoid colon $D_{0.03{\mathrm{cm}}^{3}}$	+1.3** (+0.8; +2.2)	+0.2 (−1.5; +1.6)	−0.7 (−1.3; +0.2)	−0.1 (−0.5; +2.8)

Nonetheless—and most importantly—the effect of LET_d_ optimization on plan robustness appeared relatively mild compared to the simultaneous reduction in uncertainties related to RBE model choice, observed with high statistical significance. This effect was especially prominent in potentially underdosed regions of the target, with median LEM-mMKM dose difference reduced by 9.0% and 6.3% of the prescription dose for D_95%_ and D_50%_ in the GTV respectively (*p* < 0.001).

## 4 Discussion

In the present work, LET_d_ optimization was evaluated as a potential strategy to bridge the differences between biological dose predictions made with LEM-I and HIT-mMKM in the CIRT of osteosarcoma. This is the first in-depth analysis of the impact of LET_d_ escalation on the discrepancies between LEM-I and HIT-mMKM and the first LET_d_ optimization study for this indication.

Different approaches have been used for integrating biological dose predictions from different models, most commonly to incorporate mMKM information into a LEM-based plan [28]. Generally, European centers aim to balance mMKM target coverage against historical LEM-I OAR constraints in order to minimize potential adverse effects from dose escalation. Mixed- and multi-RBE approaches utilizing both LEM- and mMKM-based optimization functions simultaneously have been suggested, with mixed-RBE optimization being based on mMKM/NIRS-mMKM target objectives and LEM-I-derived OAR constraints, and multi-RBE optimization using target objectives based on both LEM-I and mMKM/NIRS-mMKM simultaneously [29]; these, however, have not yet reached clinical implementation. In clinical experience, a common strategy is to optimize the plan using one model, recalculate the dose with the other and reevaluate the plan against its objectives, then adjust the plan manually and repeat the process until the clinical goals are reached [28], [30]. While relatively straightforward and achievable within a commercially available TPS, this approach is neither particularly time-efficient nor universal, with no concrete widely applicable techniques described in detail in literature as of now.

In this paper, boosting target LET_d_ using LET_d_ optimization has been demonstrated to result in better plan uniformity upon recalculation of originally LEM-based osteosarcoma treatment plans with mMKM and has overall lowered the discrepancies between LEM-I and mMKM's predictions. Previously, similar results were reported for large sacral chordomas, where using LET_d_ optimization resulted in significant reduction in the LEM-to-mMKM differences for D_95%_ as well as D_50%_ in the target [31].

With that in mind, the benefits of utilizing LET_d_ optimization strategies are twofold: in addition to improvements in tumor control associated with LET_d_ escalation, boosting LET_d_ can become an alternative or a time-saving addition to existing dual-RBE planning techniques. One thing to keep in mind is that for larger tumors, an additional beam is often necessary; this will inevitably prolong treatment delivery, even considering a simultaneous decrease in delivery time per beam. Hypofractionated regimens could be utilized to balance out this change and avoid losses in treatment capacity [32].

Perhaps the most important limitation of LET_d_ optimization is the potential loss of plan robustness. The conflict between LET_d_ escalation, plan uniformity and robustness is a known issue that is especially prominent in the treatment of large tumors, with only two out of these three goals typically achievable at the same time [31], [33]. The findings of the present paper confirm this trend: the addition of robust optimization on the target in LET-optimized plans likely prevented the LET_d_ from meeting the 45–55 keV/μm objectives, and literature data suggests that even much higher LET_d_ goals would have likely led to minimal target LET_d_ gains in practice [34]. Conversely, while LET_d_ optimization led to increased minimum LET_d_ and improved mMKM dose uniformity, it also slightly increased target dose variability under setup and range uncertainties as well as anatomical changes. Overall, this trade-off appears minor compared to the effect on reducing the discrepancy between RBE model predictions. Still, ideally, it should not be disregarded, and the use of various techniques such as immobilization devices (not used in the present study) or adaptive CIRT [35], [36] to mitigate the robustness losses warrants further investigation. For range uncertainties, which presented the largest concern in the current study, the use of dual- and multi-energy CT could improve stopping power ratio estimation accuracy and reduce the associated uncertainty [37], [38].

The results of the OSCAR trial revealed that pelvic location of the tumor and a GTV volume above the cohort median were associated with significantly lower overall survival compared to craniofacial localization and smaller tumor volumes [8], [26]. All the CIRT plans for the trial were originally optimized without any LET_d_ boosting strategies, and with LEM-I as the only RBE model. In larger tumors, this led to suboptimal LET_d_ distributions and, in turn, larger discrepancies between LEM-I and mMKM dose, likely resulting in an underdosage of the central portion of the tumor. Using LET_d_ optimization could help reduce these RBE-related uncertainties in the carbon ion boost and bring the dose/volume metrics for pelvic tumors closer to those of craniofacial tumors. It is important to note, however, that the CIRT boost plan only represents a part of the total dose, and other major contributing factors such as histological differences between pelvic and craniofacial osteosarcoma should be taken into account as well.

In conclusion, this study provides a proof-of-concept for the use of LET_d_ optimization to bridge the gap between LEM-I and mMKM's biological dose predictions without compromising target coverage. While the successful clinical implementation of LET_d_ optimization remains a question of balancing various sources of uncertainty with robustness being a major concern, this reduction in model-to-model variability suggests that LET_d_ boosting could be a viable strategy in cases where hybrid planning is currently used.

## CRediT authorship contribution statement

**Natalia Sycheva:** Writing – review & editing, Writing – original draft, Methodology, Investigation, Formal analysis, Conceptualization. **Katharina Kozyra:** Writing – review & editing, Investigation. **Katharina Seidensaal:** Writing – review & editing, Methodology, Investigation. **Hanna Waldsperger:** Writing – review & editing, Investigation. **Semi B. Harrabi:** Writing – review & editing, Validation, Methodology. **Jürgen Debus:** Writing – review & editing, Funding acquisition. **Andrea Mairani:** Writing – review & editing, Validation, Supervision, Methodology, Funding acquisition, Conceptualization. **Thomas Tessonnier:** Writing – review & editing, Validation, Supervision, Methodology, Conceptualization.

## Ethics declaration

Written informed consent to take part in the study and to publish the article has been obtained from all participants or their legal representatives. The privacy rights of participants have been observed.

This study was performed in compliance with relevant laws, regulatory frameworks and guidelines where the research took place. This study was conducted in accordance with Declaration of Helsinki. This study was approved by the Heidelberg University. (Approval No. S-488/2024)

## Funding statement

This work was in part funded by the Deutsche Forschungsgemeinschaft (DFG, German Research Foundation) – Projektnummer 566500585 (Andrea Mairani).

## Declaration of competing interest

The authors declare the following financial interests/personal relationships which may be considered as potential competing interests: J.D. is the CEO of the Heidelberg Ion-Beam Therapy Center (HIT) and a member of the Board of Directors of University Hospital Heidelberg. J.D. reports grants from RaySearch Laboratories AB, Vision RT Ltd., Merck Serono GmbH, Siemens Healthcare GmbH, PTW-Freiburg Dr. Pychlau GmbH and Accuray Inc. outside the submitted work; the funders had no role in the study design, data collection and analysis, the decision to publish the results, or the manuscript preparation. The remaining authors declare that they have no known competing financial interests or personal relationships that could have appeared to influence the work reported in this paper.

## Appendix A Supplementary data

Supplementary material: Additional information on LEM-I and mMKM variants, supplementary tables and figures.

## Footnotes

1 The use of Gy(RBE) here and throughout the paper refers to RBE-weighted dose as defined in ICRU Report 93.
