# Supplementary material for: Linear energy transfer optimization mitigates biological model discrepancies in carbon ion radiotherapy for pelvic osteosarcoma
Source: Phys Imaging Radiat Oncol. 2026 Oct 23;42:101113. doi: 10.1016/j.phro.2026.101113 (PMC13742323; doi:10.1016/j.phro.2026.101113)
Supplement: Supplementary file 1 [file mmc1.pdf]

Linear energy transfer optimization mitigates biological model discrepancies in carbon ion radiotherapy for pelvic osteosarcoma

Natalia Sycheva, Katharina Kozyra, Katharina Seidensaal, Hanna Waldsperger, Semi B Harrabi, Jürgen Debus, Andrea Mairani, Thomas Tessonnier

Supplementary material

Supplementary Material A. Additional information on LEM-I and mMKM variants

The Local Effect Model (LEM-I) [1,2] assumes that the biological effect of radiation can be estimated based on the microscopic radial energy deposition pattern around the particle track (“local dose”). For the survival fraction *S*, after a linear-quadratic segment, it suggests a linear dose dependency beyond a certain threshold dose D_t:

$$-\ln S = \begin{cases} \alpha_x D + \beta_x D^2 & \text{for } D < D_t \\ \alpha_x D_t + \beta_x D_t^2 + (\alpha_x + 2\beta_x D_t)(D - D_t) & \text{for } D \geq D_t, \end{cases} \tag{1}$$

where D is the dose, and α_x and β_x are the linear-quadratic parameters for the reference photon radiation.

In LEM-I, dose-averaged linear energy transfer ($\mathrm{LET_d}$) is taken into account implicitly via the local dose.

The modified microdosimetric kinetic model (mMKM) offers a distinctly different approach. It is based on the theory of dual radiation action (TDRA), where the sensitive volume of the cell is divided into subdomains and the effect of radiation emerges as a result of pairwise interaction of sublesions whose induction probability depends on the specific energy z_{1D}^* delivered in the subdomain, and the relationship between dose and cell survival fraction is linear-quadratic [3,4]:

$$-\ln S = \alpha D + \beta D^2 \; = \; (\alpha_0 + \beta z_{1D}^*)D + \beta D^2, \tag{2}$$

where α and β are the linear-quadratic parameters for the reference radiation, and α_0 is the slope of the survival curve as the LET approaches zero.

In mMKM, $\mathrm{LET_d}$ is explicitly included through the specific energy. Both the Japanese (NIRS/QST) and the HIT versions of mMKM use this formalism, but with different clinical implementations. The NIRS/QST dose prescription system involves a two-step process, where the RBE model predictions are derived based on in-vitro cell survival data (with carbon ions as reference radiation) and then scaled by a factor F_{clin} to obtain a clinical (no longer RBE-weighted) dose corresponding to past clinical experience [5].

No such clinical factor is utilized at HIT, and photons, rather than carbon ions, were chosen as the reference radiation [6]. This means that it is no longer possible to directly translate *clinical* NIRS/QST mMKM schedules to *RBE-weighted* doses in HIT mMKM. However, the use of photons as reference has an important advantage: it allows for easier comparison and combination of different radiation modalities, for example, in the context of carbon boost schedules, such as the one utilized in the OSCAR trial [7].

Typically, while LEM-I target prescriptions are more readily increased to meet mMKM objectives, constraints on doses to organs at risk remain unchanged based on the existing knowledge on LEM-I dose tolerance. As a result, at the moment, hybrid approaches are common, such as original plans being designed and optimized using LEM-I constraints and then recalculated with mMKM and adjusted to meet the mMKM objectives, which is laborious, time-consuming and heavily operator-dependent.

Table S1. Individual patient characteristics.
Initial = dose-optimized, original beam configurations; DoseOpt = dose-optimized, beam geometry identical to corresponding LET-optimized plans; LETopt = LET-optimized. Missing values in the DoseOpt column correspond to cases where the initial plan already had suitable geometry for LET optimization.

Patient	GTV volume [cm^3]	No. of beams		GTV $LET_{95\%}$ [keV/µm]		
		Initial	LETopt	Initial	DoseOpt	LETopt
**1**	463.0	1	2	27.3	33.2	46.6
**2**	818.0	3	4	29.6	30.7	49.6
**3**	1187.4	2	3	28.2	27.3	46.4
**4**	252.0	1	2	33.7	42.0	49.9
**5**	812.9	3	4	30.9	28.6	48.9
**6**	23.8	2	2	43.5	-	50.5
**7**	134.6	2	3	32.3	32.4	50.2
**8**	715.3	3	3	31.4	-	46.3
**9**	129.0	2	3	28.4	31.0	51.6
**10**	262.1	2	3	33.2	26.2	49.1
**11**	411.3	2	3	31.6	29.1	51.4
**12**	656.8	2	3	21.2	21.5	48.3
**13**	393.5	2	2	24.9	-	44.7
**14**	427.3	2	3	33.9	30.4	47.6
**15**	69.9	2	3	41.6	45.5	54.2

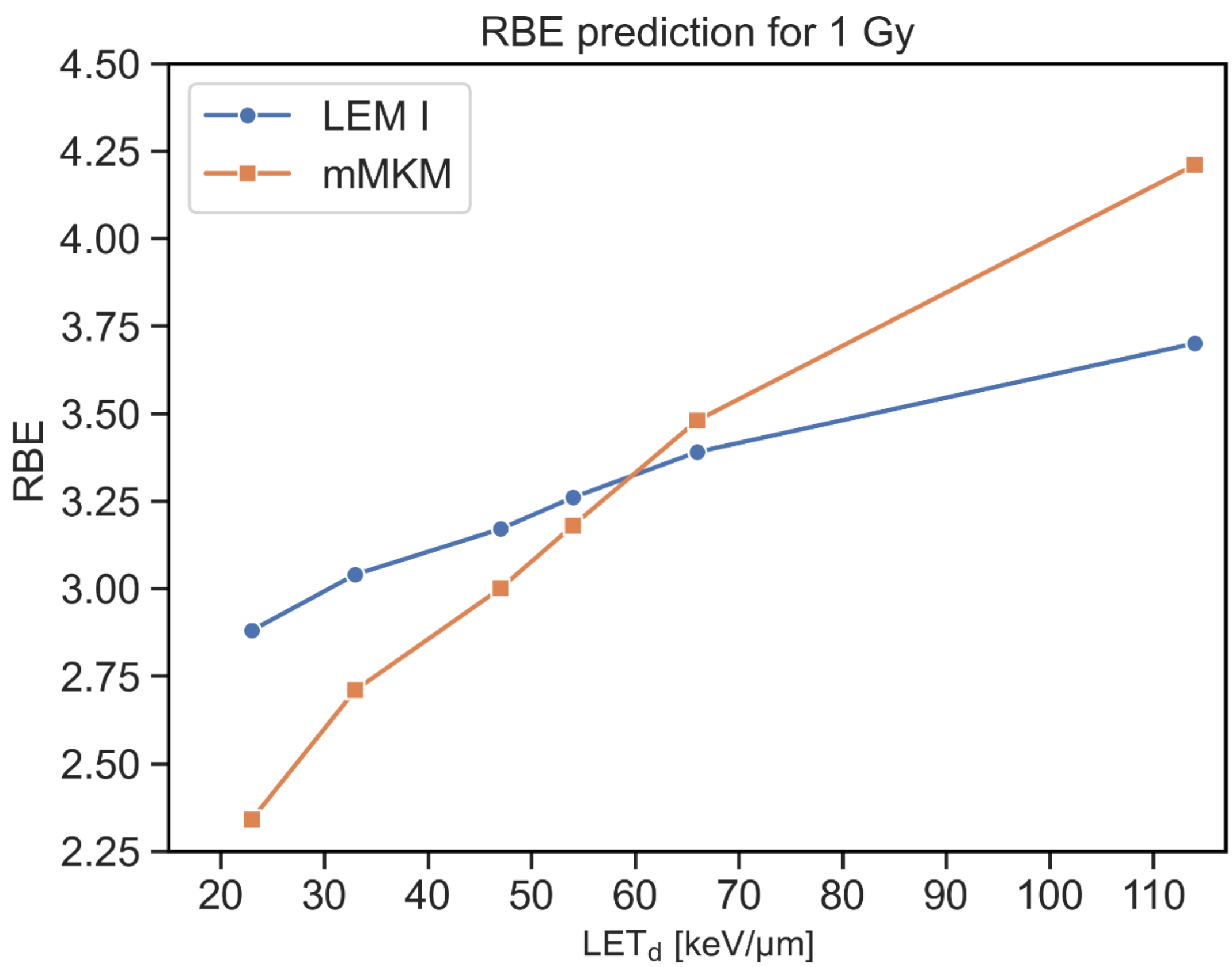

Figure S1. RBE predictions made by LEM-I (α_x = 0.1 Gy^{-1}, β_x = 0.05 Gy^{-2}, D_t = 30 Gy, r_n = 5 μm [8]) and mMKM (α_0 = 0.05 Gy^{-1}, β_x = 0.025 Gy^{-2}, $r(d)$ = 0.3 μm, r_n = 3.6 μm [6]) as a function of LET$_d$ in a 4 × 4 × 4 cm^3 water volume receiving a physical dose of 1 Gy, simulated in the treatment planning system RayStation v. 2024B.

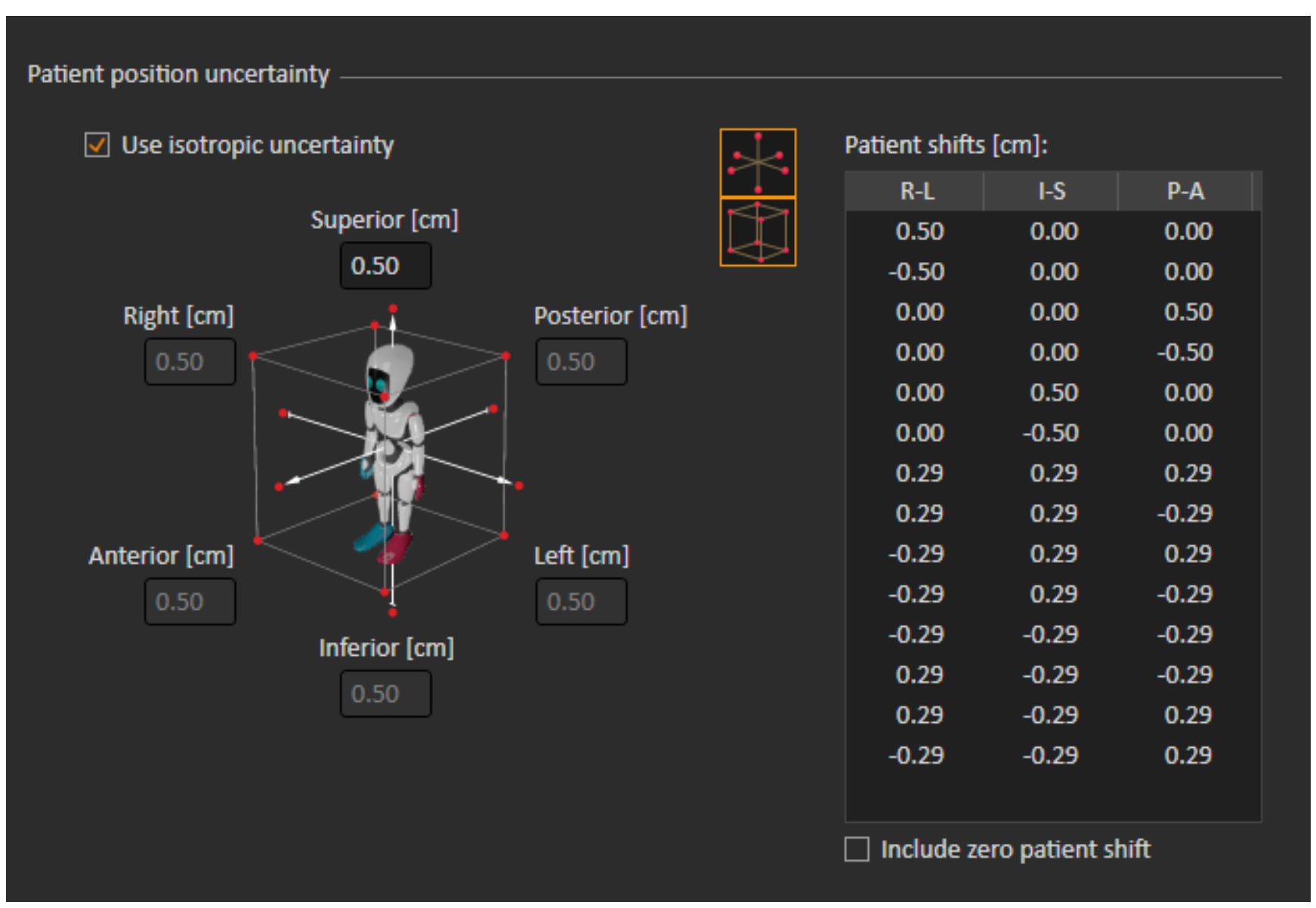

Figure S2. Geometrical positioning uncertainties used for robustness analysis within the treatment planning system RayStation v. 2024B user interface.

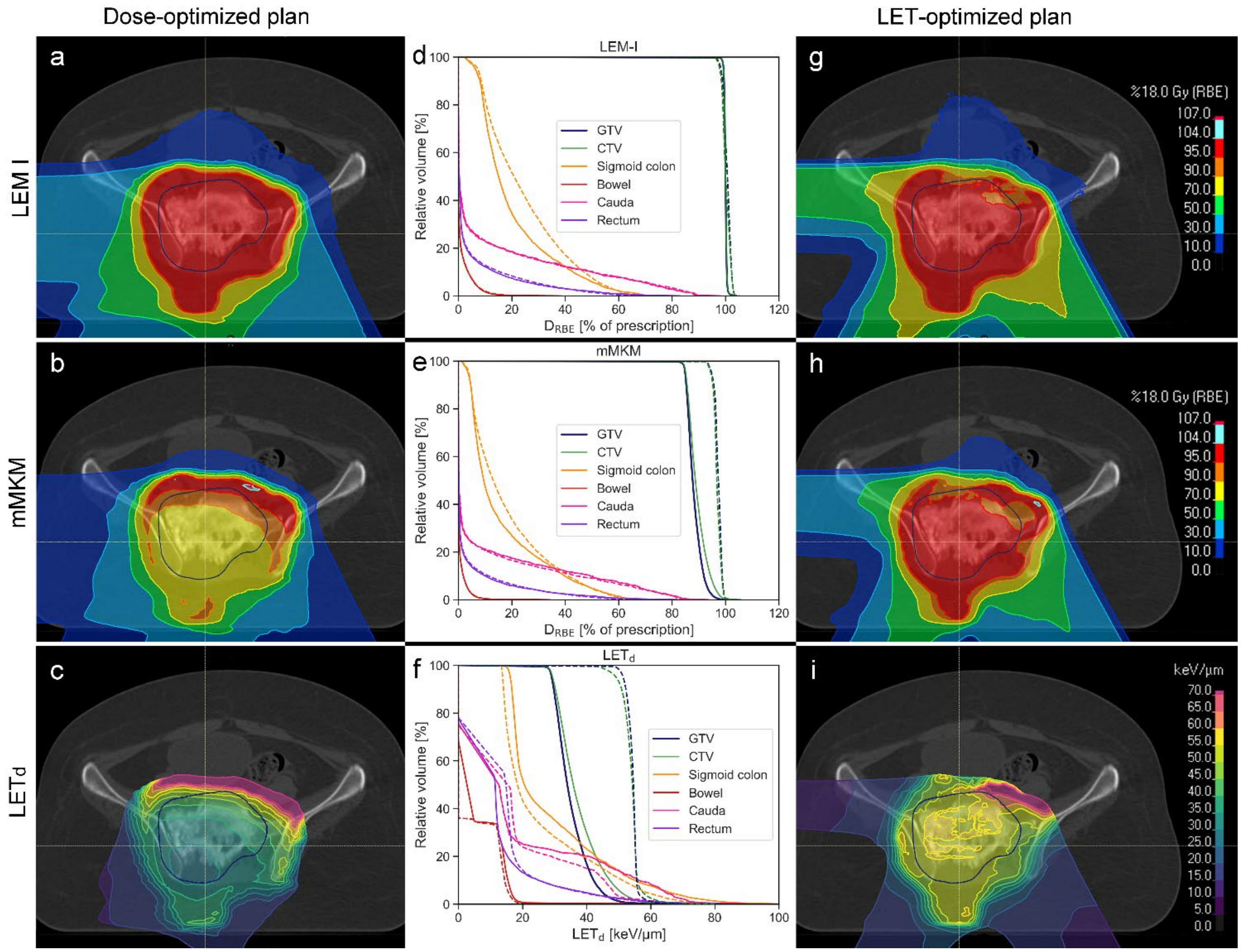

Figure S3. Axial slices of the original (LEM-I-based) biological dose, mMKM-recalculated biological dose, and LET_d distributions for the dose-optimized (a-c) and LET-optimized (g-i) plans (identical beam geometries) as well as the corresponding dose and LET_d volume histograms (d-f; solid lines: dose-optimized plan; dashed lines: LET-optimized plan) in the target and four organs at risk in a single patient case (V_{GTV} = 411.3 cm^3, cohort median). The gross tumor volume is delineated in blue.

Table S2. Summary of key target LET_d and dose metrics (median (IQR)) assessed in dose-optimized and LET-optimized treatment plans. Initial = dose-optimized, original beam configurations; DoseOpt = dose-optimized, beam geometry identical to corresponding LET-optimized plans; LETopt = LET-optimized; IQR = interquartile range. The reference prescription dose is 18 Gy(RBE).

**LET metrics**		**Initial**	**DoseOpt**	**LETopt**
GTV: $LET_{95\%}$ [keV/µm]		31.4 (28.3–33.4)	30.7 (28.0–32.8)	49.1 (47.1–50.3)
GTV: $LET_{50\%}$ [keV/µm]		38.8 (36.8–43.9)	39.4 (36.0–44.6)	54.6 (54.2–54.9)
GTV: $V_{50keV/\mu m}$ [%]		12.9 (9.3–26.5)	15.2 (9.7–28.7)	87.5 (80.4–96.8)
CTV: $LET_{95\%}$ [keV/µm]		31.3 (27.8–31.9)	30.3 (28.1–32.4)	45.6 (44.0–46.9)
CTV: $LET_{50\%}$ [keV/µm]		39.6 (38.5–45.7)	41.9 (39.1–46.4)	53.6 (52.8–54.3)
CTV: $V_{45keV/\mu m}$ [%]		32.4 (26.0–52.9)	38.5 (31.0–54.4)	96.5 (92.3–98.6)
**Dose metrics [% of prescription]**		**Initial**	**DoseOpt**	**LETopt**
GTV: $D_{95\%}$ [%]	LEM-I	98.5 (98.0–98.9)	99.0 (98.5–99.3)	98.2 (97.2–98.4)
	mMKM	85.2 (83.0–86.0)	85.6 (83.0–86.4)	93.9 (93.0–94.7)
GTV: $D_{50\%}$ [%]	LEM-I	100.0 (100.0–100.0)	100.0 (100.0–100.0)	100.0 (99.9–100.1)
	mMKM	90.0 (89.1–92.5)	90.3 (88.7–92.9)	96.8 (96.5–97.2)
GTV: $D_{2\%}$ [%]	LEM-I	102.2 (101.7–102.9)	101.7 (100.9–102.9)	102.7 (101.8–103.4)
	mMKM	100.1 (99.1–103.5)	100.3 (99.1–103.1)	101.0 (100.1–104.2)
CTV: $D_{95\%}$ [%]	LEM-I	97.4 (96.4–98.3)	98.6 (97.2–99.1)	97.8 (96.7–98.0)
	mMKM	85.3 (81.6–85.6)	85.1 (82.9–85.9)	92.4 (91.5–93.5)
CTV: $D_{50\%}$ [%]	LEM-I	100.0 (100.0–100.0)	100.0 (100.0–100.0)	100.0 (100.0–100.0)
	mMKM	90.4 (90.0–93.2)	91.5 (90.1–93.5)	96.3 (96.1–96.5)
CTV: $D_{2\%}$ [%]	LEM-I	102.5 (101.9–103.0)	102.1 (101.1–103.0)	102.8 (102.1–103.5)
	mMKM	103.5 (101.7–106.3)	104.1 (101.2–107.0)	103.3 (102.1–106.8)

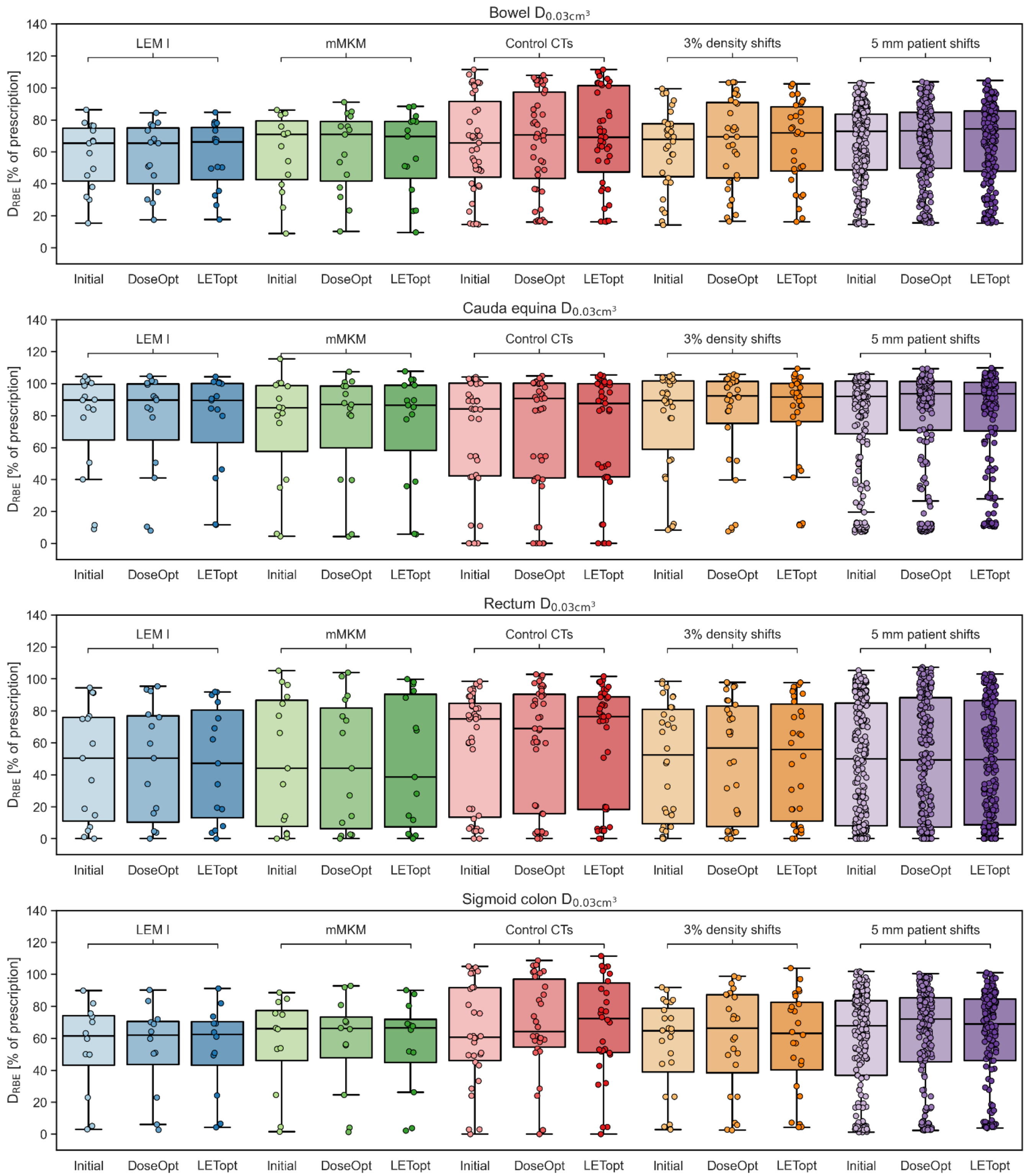

Figure S4. Evaluation of $D_{0.03\mathrm{cm}^3}$ in the organs at risk with dose-based optimization and LET optimization aimed to achieve a minimum LET_d of 50 keV/µm in the GTV and a minimum LET_d of 45 keV/µm in the CTV. Initial = dose-optimized, original beam configurations; DoseOpt = dose-optimized, beam geometry identical to corresponding LET-optimized plans; LETopt = LET-optimized. LEM-I plans were additionally evaluated with respect to control CTs acquired during treatment, 3% density shifts (2 scenarios) and 5 mm geometric shifts (14 scenarios). The reference prescription dose is 18 Gy(RBE).

References

[1] Scholz M, Kraft G. Track structure and the calculation of biological effects of heavy charged particles. Adv Space Res 1996;18:5–14. https://doi.org/10.1016/0273-1177(95)00784-C.

[2] Scholz M, Kellerer AM, Kraft-Weyrather W, Kraft G. Computation of cell survival in heavy ion beams for therapy. Radiat Environ Biophys 1997;36:59–66. https://doi.org/10.1007/s004110050055.

[3] Kase Y, Kanai T, Matsumoto Y, Furusawa Y, Okamoto H, Asaba T, et al. Microdosimetric Measurements and Estimation of Human Cell Survival for Heavy-Ion Beams. Radiat Res 2006;166:629–38. https://doi.org/10.1667/RR0536.1.

[4] Inaniwa T, Furukawa T, Kase Y, Matsufuji N, Toshito T, Matsumoto Y, et al. Treatment planning for a scanned carbon beam with a modified microdosimetric kinetic model. Phys Med Biol 2010;55:6721–37. https://doi.org/10.1088/0031-9155/55/22/008.

[5] Inaniwa T, Kanematsu N, Matsufuji N, Kanai T, Shirai T, Noda K, et al. Reformulation of a clinical-dose system for carbon-ion radiotherapy treatment planning at the National Institute of Radiological Sciences, Japan. Phys Med Biol 2015;60:3271. https://doi.org/10.1088/0031-9155/60/8/3271.

[6] Mairani A, Magro G, Tessonnier T, Böhlen TT, Molinelli S, Ferrari A, et al. Optimizing the modified microdosimetric kinetic model input parameters for proton and 4He ion beam therapy application. Phys Med Biol 2017;62:N244. https://doi.org/10.1088/1361-6560/aa6be9.

[7] Seidensaal K, Mattke M, Haufe S, Rathke H, Haberkorn U, Bougatf N, et al. The role of combined ion-beam radiotherapy (CIBRT) with protons and carbon ions in a multimodal treatment strategy of inoperable osteosarcoma. Radiother Oncol 2021;159:8–16. https://doi.org/10.1016/j.radonc.2021.01.029.

[8] Mein S, Klein C, Kopp B, Magro G, Harrabi S, Karger CP, et al. Assessment of RBE-Weighted Dose Models for Carbon Ion Therapy Toward Modernization of Clinical Practice at HIT: In Vitro, in Vivo, and in Patients. Int J Radiat Oncol Biol Phys 2020;108:779–91. https://doi.org/10.1016/j.ijrobp.2020.05.041.
